# Optical Encoding Model Based on Orbital Angular Momentum Powered by Machine Learning

**DOI:** 10.3390/s23052755

**Published:** 2023-03-02

**Authors:** Erick Lamilla, Christian Sacarelo, Manuel S. Alvarez-Alvarado, Arturo Pazmino, Peter Iza

**Affiliations:** 1 Escuela Superior Politécnica del Litoral, ESPOL, Departamento de Física, Campus Gustavo Galindo, Km 30.5 Vía Perimetral, P.O. Box 09-01-5863, Guayaquil 090150, Ecuador; 2 Facultad de Ciencias Matemáticas y Físicas, Universidad de Guayaquil, Guayaquil 090514, Ecuador; 3 Escuela Superior Politécnica del Litoral, ESPOL, Facultad de Ingeniería en Electricidad y Computación(FIEC), Campus Gustavo Galindo, Km 30.5 Vía Perimetral, P.O. Box 09-01-5863, Guayaquil 090150, Ecuador; 4 Center of Research and Development in Nanotechnology, CIDNA, Escuela Superior Politécnica del Litoral, ESPOL, Campus G. Galindo, Km 30.5 víA Perimetral, Guayaquil 090150, Ecuador

**Keywords:** machine learning, LG-beams, OAM-beams, optical encoding model

## Abstract

Based on orbital angular momentum (OAM) properties of Laguerre–Gaussian beams LG(p,ℓ), a robust optical encoding model for efficient data transmission applications is designed. This paper presents an optical encoding model based on an intensity profile generated by a coherent superposition of two OAM-carrying Laguerre–Gaussian modes and a machine learning detection method. In the encoding process, the intensity profile for data encoding is generated based on the selection of *p* and *ℓ* indices, while the decoding process is performed using a support vector machine (SVM) algorithm. Two different decoding models based on an SVM algorithm are tested to verify the robustness of the optical encoding model, finding a BER $=10^{-9}$ for 10.2 dB of signal-to-noise ratio in one of the SVM models.

## 1. Introduction

Since the study of Allen et al. [1], optical beams with orbital angular momentum (OAM) have aroused growing interest from researchers around the world due to their wavefront helical shape properties that provide a new degree of freedom for exploration of new applications in particle manipulation [2,3], image processing [4,5] and optical communications [6,7]. In this context, optical communications systems have found a possibility of exploring vortex beams properties in multiplexing [8,9] and data encoding [10,11] pathways. Concerning data encoding, OAM states to encode different data symbols are evidenced by Fang et al. [12], where OAM holography is performed by OAM selectivity in a spatial-frequency domain without a theoretical helical mode index limit. In the area of holographic encryption, Xiao et al. [13] propose a two-coding information metasurface to achieve OAM-encrypted holography. OAM encoding has also been explored in multicasting links; for instance, Shiyao Fu et al. [14] encode digital signals through the OAM free space one-to-many multicasting link. Within the same research line, data coding has also been explored experimentally as demonstrated by Willner et al. in [15], where data encoding at 20 Gb/s, using 4 possible OAM modes, is performed. High-dimensional data encoding through a hybrid OAM-radial index is also demonstrated in [14]. Optical encoding and multiplexing techniques in OAM channels for highly dispersive media have also been implemented [11], where a novel scattering-matrix-assisted retrieval technique was proposed to demultiplex OAM channels from highly scattered optical fields.

There is a lot of evidence of OAM applications, for instance, in the data encoding field in free space and fiber-based transmission channels [16,17], polarization-based [18,19] and intensity and vortices in phase-based channels [20]. However, implementation of an OAM-based encoding system requires overcoming several challenges from the point of view of information medium propagation and system detection implementation. Due to the nature of information propagation, some effects can be induced in the medium, such as absorption, scattering and turbulence, spatial distortion (amplitude and phase), modal coupling and crosstalk. Some of these effects, as in the case of turbulence and modal crosstalk, have been potentially suppressed in coding and multiplexing systems through mitigation methods [21], but for the most part, these effects constitute a great challenge [11,22]. Such challenges have captured the attention of the scientific community to focus their studies on designing more robust and flexible optical encoders and encryptors based on coding techniques that minimize noise and information distortion, while correctly maximizing the amount of data coded. In this way, the efforts to improve optical encoding systems are reflected in image recognition methods for encoded data, as in the case of [23], where an index modulation is implemented for OAM states with a uniform scheme circular array (OAM-UCA) to build low-intensity parity coding to improve error performance and transmit additional bits of information. Incoherent detection methods have also been implemented for data decoding [24], where an image information transfer method based on petal-like beam lattices for coding is used. In this case, a decoding system works directly with the identification of the intensity patterns captured. Another example of an image-based method is presented in [20] that employs the amplitude and the phase of an optical field into a phase-only hologram to control spatial transverse modes for data symbol mapping. A similar study can be found in [25] that uses an OAM array for a free-space communication encoding/decoding link with 625 states. A proposal for OAM light encoding in magnets has also been developed in [26], where the possible sub-wavelength magnetic phenomena induced by a vortex beam and their applications in the generation of topological defects in chiral magnets is discussed. Although the aforementioned studies show the feasibility of encoding systems based on OAM modes, OAM does not increase the amount of information, nor does it exceed the multiple-input multiple-output (MIMO) transmission of current standards in optical communications [7,27]. In fact, the number of spatial modes available for data encoding is limited by the space-bandwidth product of a given optical system [27,28]. A solution to this problem is to use all spatial degrees of freedom offered by OAM modes. A commonly used OAM beam for this purpose is a Laguerre–Gaussian (LG) beam [7,17], which provides eigen-modes dependent on both radial (*p*) and azimuthal (*ℓ*) indices, being able to use the superposition of modes to increase the number of encoding data in a limited system. On the other hand, a decoding system (which is generally based on image detection and classification) can present strong signal distortion (both in the intensity profile and in the phase distribution) due to optical alignment, turbulence and scattering [29]. Recently, convolutional neural networks (CNNs) and machine learning techniques have been implemented in optical coding systems as an alternative solution for image detection and classification [30,31,32]. High-resolution recognition techniques based on deep learning to encode data in spatial modes have already been implemented [33]. The deep-learning-based approach has also been used to recover the sparse data from multiplexed OAM channels independent of phase information [34]. Although these studies demonstrate the feasibility of encoding systems based on OAM modes as well as various methods implemented for data decoding, there is still a gap concerning image detection and classification methods in decoding due to degradation effects that the medium induces in the transmitted signal, which brings the motivation for this research work.

Motivated by previous statements, this paper proposes a comprehensive optical encoding–decoding system based on the intensity profile generated by a coherent superposition of two OAM-carrying Laguerre–Gaussian (LG) modes and a machine learning detection method. In the encoding process, an intensity profile for data encoding is generated based on the selection of *p* and *ℓ* indices of LG beams, while the decoding process is performed using support vector machine (SVM). Different from other existing encoding systems that require the additional extraction of phase information, this paper proposes a novel optical encoder based on the number of spatial modes carrying data symbols increased in a limited optical system. Moreover, the proposed optical encoding model opens a pathway to a stable image detection and classification system based on machine learning that only uses the intensity profile for target modes. As a result, the main contributions of this paper are: (1) a comprehensive design of a coherent optical encoding system based on the superposition of LG modes carrying OAM that is independent of phase information and (2) a robust decoding system based on intensity profile recognition using the machine learning SVM method. Section 2 presents the concept and operating principle of the optical encoder. In Section 3, the SVM-based decoding method for image recognition and classification is explained in detail. In Section 4, a case study for a 4-bit coding system with different types of noise is considered to validate the robustness of the proposed encoder. Finally, Section 5 and Section 6 exhibit the results and conclusion, respectively.

## 2. Concept and Principle of the Optical Encoding Model

**Figure 1:**
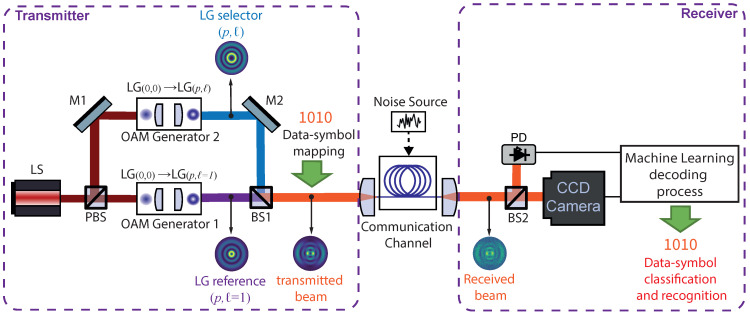
Concept and proposed setup of an optical encoding model. LS: laser source; PBS: polarization beam splitter, M1,2: mirror; BS1,2: beam splitter; PD: photodetector.

The schematic setup of the conceptual art of this proposed optical encoding model is illustrated in Figure 1. On the transmitter side, an optical system based on a Mach–Zehender interferometer is used to generate a coherent combination of two Laguerre–Gaussian (LG) beams carrying orbital angular momentum (OAM). A laser source provides a coherent fundamental Laguerre–Gaussian (LG00) beam in free space that is launched to a polarization beam splitter (PBS) to control relative power between the reference and the selector arm. Both arms will go through an OAM generator to convert a fundamental LG(p=0,ℓ=0) mode to a higher-order LG(p,ℓ) mode carrying OAM. The reference arm is converted to an LG(p,ℓ=1) mode (via OAM Generator 1 in Figure 1), while selector arm is converted to an LG(p,ℓ) mode carrying OAM (via OAM Generator 2 in Figure 1). Since the topological charge *ℓ* and the radial index p at the LG(p,ℓ) selector beam can be properly selected to generate the intensity pattern for the optical encoder, this mode index will be the code-key numbers associated with the data symbol. The reference and selector arms are combined through a beam splitter (BS1), and the intensity profile of this superposition will be the pattern corresponding to a unique data symbol associated with (p,ℓ) combination. After encoding, the transmitted output beam is transferred to a communication channel in which different noise sources will be added in order to affect the signal. On the receiver side, the received beam is decoded by a machine learning process using an SVM-based method for image recognition and classification.

For the generation stage of the intensity pattern that will be used on the transmitter side, the mathematical formulation of the Laguerre–Gaussian beams [35,36] has been used, which is characterized by two indices (p,ℓ) corresponding to radial and azimuthal distribution, respectively. The optical field of an LG(p,ℓ) mode can be represented by:

(1) $$\begin{array}{cc} \; & LG_{p,\ell }\left(r,\theta ,z\right)\\ \; & =\sqrt{\frac{2p!}{\pi (p+|\ell |)!}}\frac{1}{w(z)}{\left(\frac{r\sqrt{2}}{w(z)}\right)}^{\left|\ell \right|}L_{p}^{\left|\ell \right|}\left(\frac{2r^{2}}{w^{2}\left(z\right)}\right)\\ \; & \exp\left(\frac{-r^{2}}{w^{2}\left(z\right)}\right)\exp\left(\frac{ik_{o}r^{2}z}{2\left(z^{2}+z_{R}^{2}\right)}\right)\exp\left(\Phi \left(z\right)\right)\exp\left(i\ell \theta \right) \end{array}$$

where w(z) is the beam width, $z_{R}$ is the Rayleigh range and Φ(z) is the Gouy phase. $L_{p}^{\left|\ell \right|}$ are the generalized Laguerre polynomials, and (r,θ,z) represents the cylindrical coordinate. Then, the superposition of two LG modes carrying OAM [37] can be expressed as:

(2) $$u\left(r,\theta ,z\right)=LG_{p^{\prime },\ell^{\prime }}\left(r,\theta ,z\right)+LG_{p,\ell }\left(r,\theta ,z\right)$$

The first term in Equation (2) describes the reference field, while the second term represents the optical field that acts as a selector. As mentioned, for the optical encoder presented in this work, the LG(p,ℓ=1) mode will be used as the $LG_{p^{\prime },\ell^{\prime }}\left(r,\theta ,z\right)$ reference beam, while the selector beam $LG_{p,\ell }\left(r,\theta ,z\right)$ will be a previously selected $LG_{p,\ell }$ mode. The same radial index *p* has been chosen for both reference and selector beam in order to simplify the design of the encoder. An intensity profile of u(r,θ,z) is associated with a data-bit sequence according to the (p,ℓ) parameters used in the selector beam generation. Since OAM beams have twisted helical phase fronts, often characterized by the azimuthal index *ℓ* (also named topological charge), while propagating [11], the intensity profile will be most affected in rotations along the propagation axis, without significant changes in the intensity pattern. Additionally, the property of orthogonality between LG modes allows the resulting intensity pattern to be unique for each data symbol [38].

## 3. SVM-Based Decoding Method for Image Recognition and Classification

The proposed optical encoding model takes as input a 4-bit code defined by the variable *X*. In addition, a signal noise ratio (SNR) is used to emulate the noise in the communication channel that is given in decibels. The encoding starts with the definition of the variables $\ell_{1}$, $\ell_{2}$ and *p* to establish the intensity profile, which is executed by the function selectCode. Then, two different intensity profiles are generated using the mathematical formulation given in Equations (1) and (2) and declared in functionLG. This is followed by the representation of the intensity profile in terms of Cartesian coordinates x,y and the intensity of the resulting beam profile declared in variable *I*. Next, with a view to emulate a real communication, signal noise is added to the transmitted intensity profile stated in the function addNoise. Later, the extractHOGFeaturesFromIntensity function is used to extract useful patterns for information recognition through histogram of oriented gradients (HOG) detection [14]. Finally, the function predict, which is based on a linear regression model, is used as a 4-bit classifier through a multiclass error-correcting output codes (ECOC) model using SVM binary learners. For more details about the followed process, Algorithm 1 is presented. As the process involves training procedures, the decoding processing at the receiver side of the encoder is based on SVM. More details on this SVM algorithm can be found in the Appendix A.
**Algorithm 1** Pseudocode for decoding processing using an SVM–ECOC model1:**Input** X, SNR2:**Transmission side:**3:[$\ell_{1},\ell_{2},p$] = selectCode(X) ▹ *Give $\ell_{1},\ell_{2},$ and p values according to Code Table*4:[$x_{1},y_{1},z_{1}$] = $functionLG(\ell_{1},\theta_{o}=0,\lambda ,z=0,p)$ ▹ *Generate 1st Intensity Profile accord. Equation1*5:[$x_{2},y_{2},z_{2}$] = functionLG($\ell_{2},\theta_{o}=0,\lambda ,z=0,p$) ▹ *Generate 2nd Intensity Profile accord. Equation1*6:$x\leftarrow x_{1}$7:$y\leftarrow y_{1}$ ▹ *Generate Value for Cartesian coordinates*8:$I\leftarrow z_{1}+z_{2}$ ▹ *Superposition of Intensity Profile accord. Equation2*9:**Communication Channel:**10:n=addNoise(I,SNR) ▹ *Add Noise to the intensity in order to simulate real communication signal*11:**Receiver Side:**12:TestFeatures=extractHOGFeaturesFromIntensity(n) ▹ *Extract HOG features for the Intensity profile with noise*13:Y=predict(classifier,testFeatures) ▹ *Use model from SVM-ECOC Multiclass Training*14:**Output** Y

## 4. Case Study

**Figure 2:**
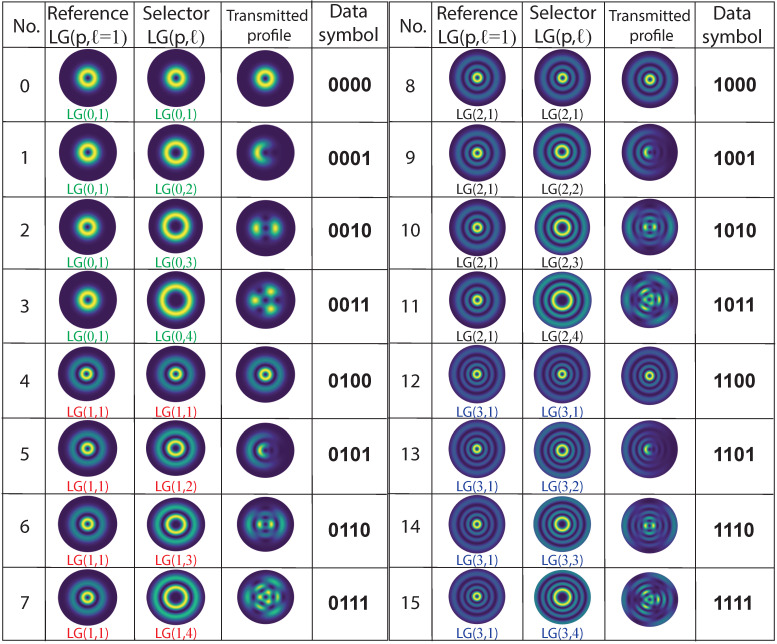
Data symbol set based on a 4-bit data symbol for the case study presented.

As mentioned in the operating principle of the proposed optical encoding model, each data symbol is mapped to a corresponding u(r,θ) profile according to the selected modal indices *ℓ* and *p* in the selector beam. Since the reference beam is restricted to the $LG(p^{\prime },\ell^{\prime }=1)$ mode, the alphabet for possible data symbols within a discrete time window can be calculated as $log_{2}N$ with $N=n_{\ell }n_{p}$, where $n_{\ell ,p}$ represents the number of *ℓ* and *p* indices used in the selector arm, and *N* represents the different data symbols that can be encoded as *N* -ary numbers: 0,1,…(N−1) [14]. For validation purposes, a data symbol code based on a 4-bit data symbol (N=16) is designed, which is associated with the resulting intensity profile according to the selection of the (p,ℓ) combination, as shown in Figure 2. In the simulations presented in this work, LG beams with wavelength λ=1550 nm, fundamental beam width $w_{0}=100\lambda$ and a propagation distance z=200λ have been considered. This table shows all possible combinations of data symbols and the normalized intensity profiles of the reference, selector and the transmitted beam for data mapping.

Since the proposed optical encoding model operates based on the Mach–Zehnder [39,40] interferometric method on the transmitter side, and an image-based detection system on the receiver side, the following OAM generation methods must be considered for an experimental implementation: For the experimental generation of OAM modes, it is common to use mode converters composed of several cylindrical lenses, which can convert high-order Hermite–Gaussian beams into high-order Laguerre–Gaussian beams. However, mode converters are limited to a specific order Hermite–Gaussian beam, which needs to be generated by certain technical means as presented in [2]. The size of the mode converter is large, which presents strict requirements for the relative position and angle of the cylindrical lens. Typical mode converter configurations can be founded in [41]. Another alternative of mechanism for the OAM generation mode is the employment of a spatial light modulator (SLM) [11] that uses configurations based on changing modulation patterns loaded into the spatial light modulator. This can be achieved with a laser that can achieve various OAM beams with different output degrees. However, it is important to consider that under current technical conditions, the reflectivity of liquid crystal spatial light modulators is from 60 % to 90% [11].

## 5. Results

The performance of the proposed optical encoding model is measured in terms of signal degradation due to the addition of noise and the bit error rate (BER) presented by the system. It is known that accuracy and precision of an optical encoder depend on detection method and robustness of the SVM training algorithm used [42,43]. In this context, to validate the influence of noise on transmission and therefore measure the degree of degradation and signal detection, a combination of RIN and AWGN noises has been used as channel noise for all detection and classification cases. The value of α for RIN has been established by a factor of 0.5 of the uniform random distribution, while for AWGN the mean μ=0 and signal–noise ratio (SNR) levels have been established at 36 dB (low), 30 dB (medium) and 24 dB (high) that are typical noise levels in optical communication systems [44,45].

**Figure 3:**
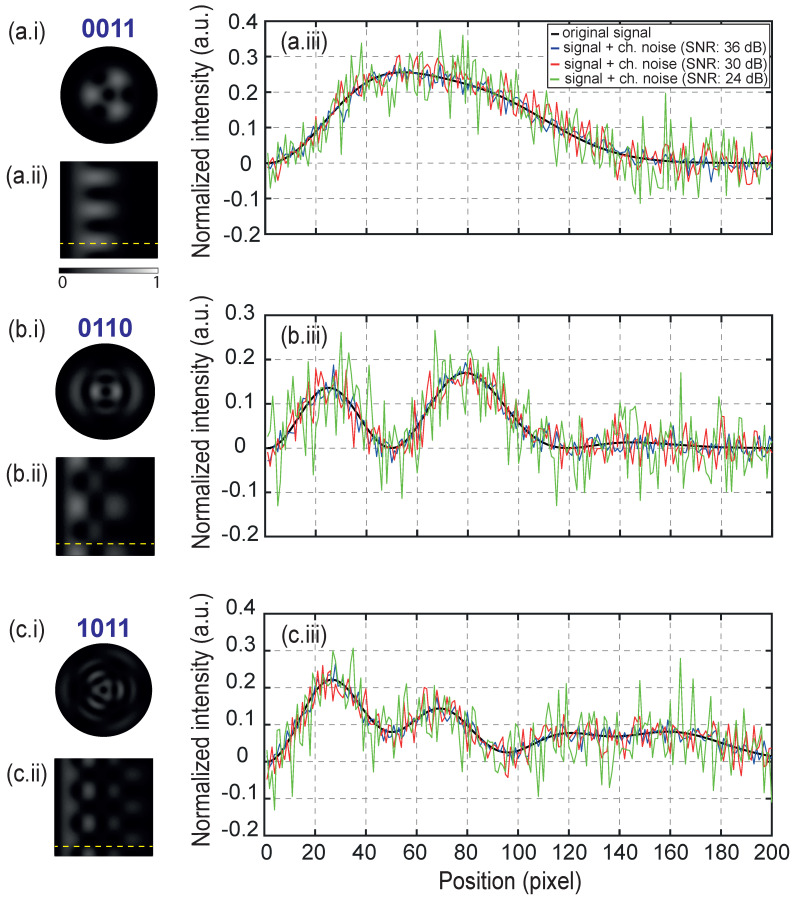
Different data symbols and their corresponding normalized intensity curves: (**a.i**) data symbol 0011; (**b.i**) data symbol 0110; (**c.i**) data symbol 1011; (**a.ii**,**b.ii**,**c.ii**) linear transformation of (**a.i**,**b.i**,**c.i**); (**a.iii**,**b.iii**,**c.iii**) normalized intensity curve corresponding to a pixel array of a 2D image (dotted yellow line) with different channel noise levels.

To understand how the combination of these noises affects the transmitted signal, three different data symbols are presented in Figure 3: 0011 (Figure 3a.i), 0110 (Figure 3b.i) and 1011 (Figure 3c.i) with their corresponding 2D linear transformations of 200 × 200 pixels (computational burden), Figure 3a.ii,b.ii,c.ii. With a view to show the impact of the noises in the transmitted signal, the horizontal position arrangement for pixel 50 of the vertical position (x,50) has been chosen for display purposes, which is presented as a yellow dotted line in Figure 3a.ii,b.ii,c.ii. As a result, the normalized intensity curve for such an array is presented in Figure 3a.iii,b.iii,c.iii.

The normalized intensity curves show the original transmitted signal in a black curve, the received signal with the same α for each case (α = 0.5 for RIN) and with low level channel noise in a blue curve, medium level in a red curve and high level in a green curve. The results of Figure 3 reveal that despite observing distortion in the signal due to the addition of noise for levels greater than 24 dB of SNR, the SVM–ECOC model allows each image to be correctly classified and recognized, with a percentage of 100% recovery for each data symbol. For this reason, a computed BER measurement for values less than 24 dB of SNR is necessary to validate the robustness of the optical encoding model at much more critical noise levels.

**Figure 4:**
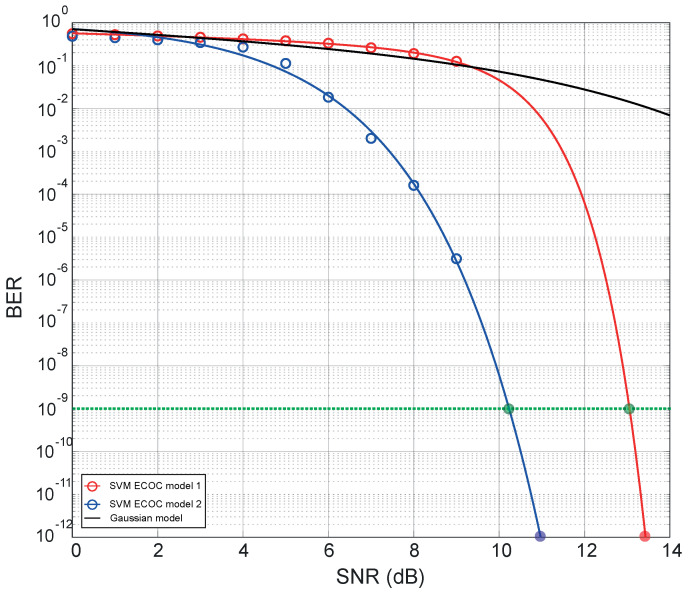
Computed BER for each SVM-ECOC model as a function of SNR for critical noise levels (from 0 to 14 dB).

An end-to-end performance measure for data transmission is BER, which quantifies the reliability of an entire coding system from “input bits” to “output bits”, including the behaviour of all components and elements between the transmitted signal and the received signal in addition to considering the path of the signal in the middle [46,47]. BER is mathematically defined as the relation between the number of bit errors and the total number of bits [48], which expresses the probability of a bit error. The machine learning model for prediction, recognition and classification of images on the receiver side of the proposed optical encoder is based on the SVM–ECOC multicast algorithm, which can be modelled with binary combinations of each class (one-vs.-one) or with binary combinations of one to multiple classes (one-vs.-all) [49,50], so the BER measurement for each machine learning model becomes a reliable metric of confidence level at the receiving point. To calculate the BER as a function of SNR at the receiver end using the SVM-ECOC model, the training model (one- vs. -one or one- vs. -all) is first created based on the data set of 4-bit symbols (see Figure 2). Then, the algorithm is trained with 750 images at different SNR levels (from 12 dB to 36 dB in steps of 6 dB) in the received signal, to test the functionality of the model at these noise levels. Once the functionality of the SVM model has been verified through the previous training, the images are processed with the model. For image processing, a database consisting of 10,000 images for each 4-bit data symbol combination (between 0000 and 1111) was used, resulting in a total of 160,000 processed images. The HOG features are extracted from each of these images to predict the combination of bits corresponding to each image, using the model. Finally, after each prediction, the acquired combination is compared with the original combination, and then the BER is calculated. Figure 4 shows the computed BER points as a function of signal-to-noise ratio (SNR) from 0 to 14 dB in steps of 1 dB for the two proposed SVM-ECOC models: the multicast one- vs. -one algorithm (Model 1) in the red curve and the Multicast one- vs. -all algorithm (Model 2) in the blue curve.

Since the standard maximum BER for most optical systems is $10^{-9}$ [51], and for applications in optical communications the maximum BER range is in the range $10^{-9}$ to $10^{-12}$ [52], the adjustment curve for each model is also shown in Figure 4 in order to predict noise levels for these values. The BER curve for Model 1 reaches BER $=10^{-9}$ for 12.8 dB of SNR (see green line in Figure 4), and BER $=10^{-12}$ for 13.4 dB of SNR. For the case of Model 2 (blue curve in Figure 4), BER $=10^{-9}$ for 10.2 dB of SNR (see green line in Figure 4), while for a BER $=10^{-12}$ for 10.9 dB of SNR. Additionally, for comparison purposes, a BER estimation curve assuming a probability of error with a Gaussian random variable [53] is also shown in a black curve. It is observed that both SVM–ECOC models have better performance compared to the simplified Gaussian BER model in terms of noise levels, highlighting that Model 2 has a better probability of error compared to Model 1, with a difference of 2.65 dB of noise level for BER $=10^{-9}$. Note that since the channel noises used in the simulation are AWGN and RIN, the bit errors generated in this case study are directly due to signal degradation by these types of noise. This fact is observed in the results of Figure 4 for each model, indicating that for an SNR level greater than 9 dB, the bit error probability is below 10% for Model 1 and below 0.0001% for Model 2.

## 6. Conclusions

A comprehensive design of an optical encoding model based on the coherent superposition of two LG beams with OAM is proposed for the generation of a coding system independent of phase information. The proposed approach employs an SVM-ECOC algorithm machine learning that enables image prediction, recognition and classification. To verify the robustness of the proposed optical encoding model, a data symbol code based on a 4-bit data symbol is designed, which is associated with the intensity profile according to the (p,ℓ) combination. A channel noise made up of the RIN and AWGN is added to the images generated in the encoding stage to emulate a real environment. In order to identify each data symbol, two different algorithms based on an SVM-ECOC model are used. The efficacy of the proposed approach is validated through BER measurements. The results reveal that the proposed algorithms are able to recognize the data symbol set with a degree of confidence greater than 90% for noise levels up to 9 dB in both models. Even though both models present high efficiency, the Multicast one-vs.-all model (Model 2) presents the best BER curve between the two models studied, with a BER $=10^{-9}$ for 10.2 dB of SNR.

The proposed encoding model can be employed on optical free-space (OFS) data links, which according to the state of the art, such encoding potentially increases data capacity for wireless systems and satellite communication systems [25,54]. These systems present typical link distances between 1 km and 143 km (verified experimentally), for 532 nm, 633 nm and 1550 nm of operating wavelength and a range between 150 Mbps and 200 Gbps of data rate [11], which complicates the data transmission. However, the proposed optical encoding model can be a solution as it can be used over optical fiber links as evidenced in [25], in which an optical encoding system based on OAM beams has been implemented for data transmission at 80 Gbps using 5 km few mode fibers (FMF) to data transmission at 640 Gbps using 18 km of ring-core-fiber. On the other hand, some constraints must be considered when choosing the type of encrypted data transmission channel. For free-space links, atmospheric turbulence can cause a random phase and intensity distortion on the transversal beam profile [55], which can be quantified by the refractive index structure constant $C_{n}^{2}$ that has typical values between $10^{-17}$ m${}^{-2/3}$ and $10^{-13}$ m${}^{-2/3}$. According to Allen et al. [1], the Rytov variance is an adequate indicator to quantify turbulence fluctuations in OFS links, since this is related to $C_{n}^{2}$ and the propagation distance. Also demonstrated in [56], for a link with low power fluctuations (low Rytov variance), the recommended propagation length should be less than 10 km, and for greater distances, the use of mitigation methods such as adaptive optics beam shaping is recommended [57]. Focusing on optical fiber links, the fundamental limitations lie in the type of fiber used for the transmission channel. The use of few mode fibers (FMFs) or the use of micro-structured fibers is necessary to excite OAM modes within the fiber as evidenced in [25].

For future research, it is relevant to mention that the number of circular fringes in the intensity profile of an LG mode are directly related to the index *p*, while the spatial distribution of these fringes is related to the index *ℓ*; therefore, the number of bits can be extended to more than 4-bits for the case where ℓ≥5. This fact opens new opportunities for the development of advanced encoding systems.

## Figures and Tables

**Figure A1:**
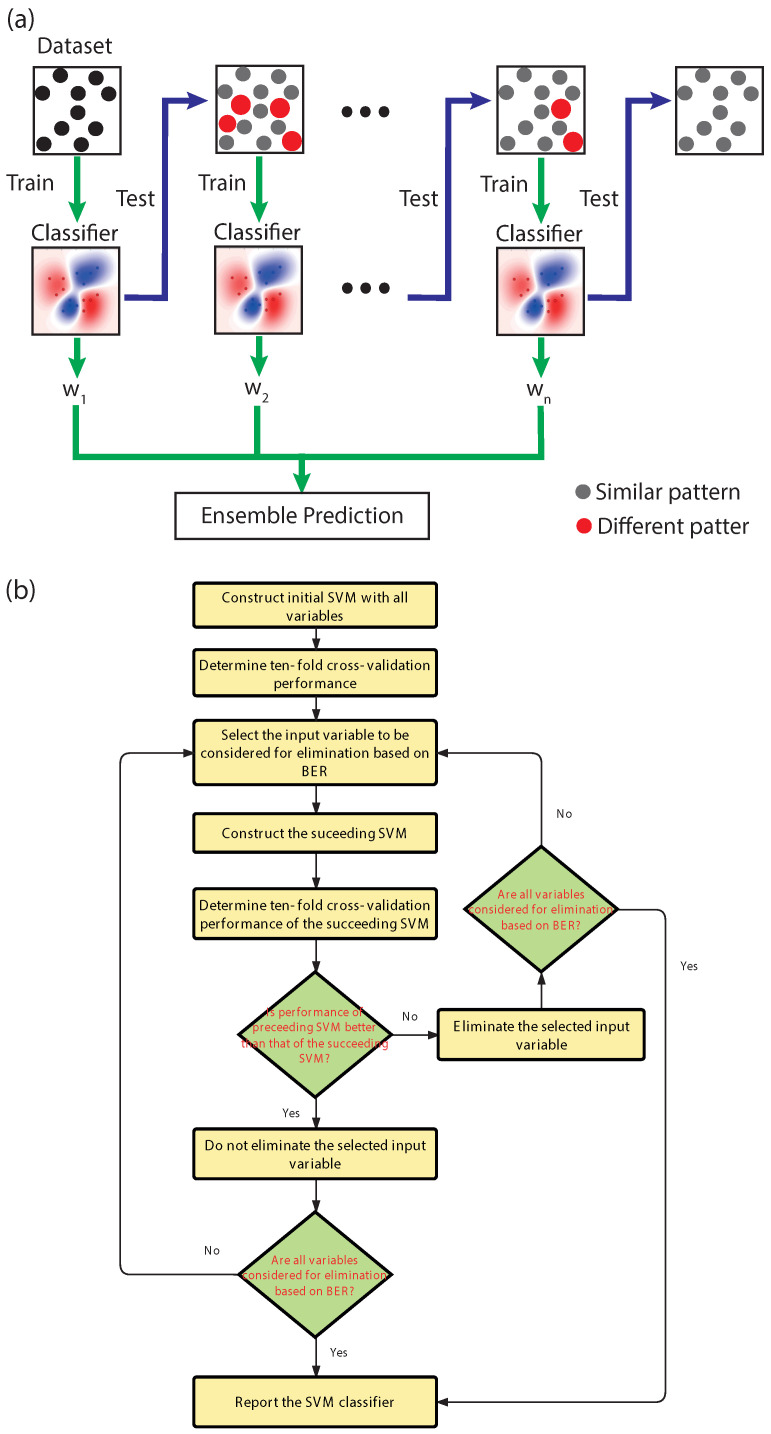
(**a**) Flow diagram of an SVM algorithm. The ensemble classifiers consist of a set of weak classifiers. The weights (wn) of the incorrectly predicted points are increased in the next classifier. The final decision is based on the weighted average of the individual predictions; (**b**) flowchart of the application of the support vector machine (SVM) algorithm in the decoding processing.

## Data Availability

Not applicable.

## Appendix A. Support Vector Machine Algorithm

As the process involves training procedures, the decoding processing on the receiver side of the encoder is based on support vector machine (SVM). SVM is a machine learning algorithm that employs the concept of the kernel function to map the data in a different dimensional space, such that the information is grouped according to similar attributes. The algorithm takes as input the raw data, which is classified depending on the kernel function. Then, the data are saved and compared with the original figure to identify similar patterns that are used for image identification. This process is repeated until the maximum number of iterations *n* is reached, as shown in Figure A1a. As a result, a simplification of complex nonlinear decision boundaries is obtained to derive in a linear dimensional space [58]. Mathematically, the characterization is driven by the kernel that can take the form as presented in Table A1. For a better understanding, a flowchart of SVM is presented in Figure A1b. The kernel used in the SVM algorithm for the proposed optical encoding model is the basis function (Gaussian).

**Table A1:** Brief description of the kernels that are used in the different types of SVM algorithms.

Type of SVM	Kernel	Description
Base function (Gaussian)	$K\left(x_{1},x_{2}\right)=e^{-\frac{{∥x_{1}-x_{2}∥}^{2}}{2\sigma^{2}}}$	Learning of one class, where σ represents the width of the kernel
Linear	$K\left(x_{1},x_{2}\right)=x_{1}^{T}x_{2}$	Learning of two classes
Polynomial	$K\left(x_{1},x_{2}\right)={\left(x_{1}^{T}x_{2}+1\right)}^{\rho }$	ρ is the polynomial degree
Sigmoid	$K\left(x_{1},x_{2}\right)=\;\tanh\left(\beta_{0}x_{1}^{T}x_{2}+\beta_{1}\right)$	The kernel is determined by specific $\beta_{0}$ and $\beta_{1}$
